# Education of the People by Physicians

**Published:** 1881-10

**Authors:** 


### Education of the People by Physicans.

There might be written a long chapter on the duty which physicians owe to the public as well as to their profession, to educate their patients and others within the circle of their influence, in various matters involving the interests of both parties. A general knowledge of the conditions of the body in health and disease, and of the nature of medicinal agents, tends greatly to fortify the mind against the delusion and imposture. The deserving physician is always best appreciated among intelligent people. As educators on medical topics newspapers are failures, or something worse. Most of the education people receive from newspapers is contained in quack advertisements. The balance consists in such sensational stories as can be picked up regarding mad-stones and blood-stones and similar superstitions and marvels. As a rule, newspapers are practically hostile to the profession. Physicians do not advertise, and quacks do. This determines the comparative value of the two classes in the estimation of newspapers. This is the reason why certain of the San Francisco newspapers are so opposed to the medical law. There are also certain quasi-medical journals, edited by half-educated men with lopsided brains, full of notions and whims, bent on gaining notoriety by what might be called scientific monkey-capers. In justice to themselves, if for no other reason, physicians should conteract the errors of such false educators by teaching persons with whom they come in contact the rudiments of medical science—inoculating them, as it were, against infection from error.—*Pacific Medical and Surgical Journal.*

